# Observational evidence for detrimental impact of inhaled ozone on human respiratory system

**DOI:** 10.1186/s12889-023-15902-6

**Published:** 2023-05-23

**Authors:** Jiaying Lu, Ling Yao

**Affiliations:** 1 State Key Laboratory of Resources and Environmental Information System, Institute of Geographic Sciences and Natural Resources Research, Chinese Academy of Sciences, Beijing, China; 2 Jiangsu Center for Collaborative Innovation in Geographical Information Resource Development and Application, Nanjing Normal University, Nanjing, China; 3 University of Chinese Academy of Sciences, Beijing, China

**Keywords:** Inhaled ozone, Respiratory disease, Convergent cross mapping method, Causative influence

## Abstract

The detrimental influence of inhaled ozone on human respiratory system is ambiguous due to the complexity of dose response relationship between ozone and human respiratory system. This study collects inhaled ozone concentration and respiratory disease data from Shenzhen City to reveal the impact of ozone on respiratory diseases using the Generalized Additive Models (GAM) and Convergent Cross Mapping (CCM) method at the 95% confidence level. The result of GAM exhibits a partially significant lag effect on acute respiratory diseases in cumulative mode. Since the traditional correlation analysis is incapable of capturing causality, the CCM method is applied to examine whether the inhaled ozone affects human respiratory system. The results demonstrate that the inhaled ozone has a significant causative impact on hospitalization rates of both upper and lower respiratory diseases. Furthermore, the harmful causative effects of ozone to the human health are varied with gender and age. Females are more susceptible to inhaled ozone than males, probably because of the estrogen levels and the differential regulation of lung immune response. Adults are more sensitive to ozone exposure than children, potentially due to the fact that children need longer time to react to ozone stress than adults, and the elderly are more tolerant than adults and children, which may be related to pulmonary hypofunction of the elderly while has little correlation with ozone exposure.

## Introduction

Ground-level ozone (O_3_) is the secondary pollutant produced by photochemical activity of nitrogen oxides and volatile organic compounds in the troposphere of the earth’s surface [1]. It has been proved that ozone at ground level has become the second ranked air pollutant in China [2], and ground observations show that in warm seasons, ozone even becomes the primary air pollutant. Ozone could cause respiratory diseases, such as asthma, pneumonia, chronic obstructive pulmonary disease (COPD), etc., through previous epidemiologic and experimental studies [3–8]. A regional study in Suzhou, a Chinese city with high ozone pollution [9], suggests that the mortality from respiratory diseases is correlated to the maximum mean concentration of ozone within 8 h and the maximum concentration of ozone within 1 h. Time-series studies also have indicated that the risk of respiratory hospitalizations or emergency visits would increase with short-term exposure to ozone [10]. It has been revealed that an approximately 0.2-0.6% significant increase in the risk of dying is associated with a per 0.01 mg m^-3^ increase in ambient ozone within 8 h, based on the meta-analysis of long-time data since 1996 [11]. As has been widely recognized, air pollution affects human health differently in different regions. Some regional studies demonstrate that inhaled ozone is associated with hospital admissions for respiratory diseases, according to Generalized Additive Models (GAM) [12–14]. Ozone has a clearly accumulative effect on respiratory disease mortalities [2] and it has a significant impact on COPD with wide temporal and spatial variation [15]. Some other studies demonstrate a weak or non-significant connection between ozone and respiratory diseases [11, 16, 17]. Consequently, respiratory disease is not consistent with ozone concentration observed at fixed sites well, and the effects of ozone on public health have seldom been evaluated independently from other air pollutants [16]. Generalized Additive Models could fail in these data, leading to a large number of observations and clinical data being excluded to avoid a possible effect bias of the ambient ozone on respiratory disease. Therefore, a new method (i.e., Convergent Cross Mapping, CCM), specifically designed for detecting causality relationship from pair of empirical time series data in nonlinear dynamical ecosystems [18], was used to address this problem.

Shenzhen, the first special economic zone of China with the highest population density (approximately 6000 persons per km^2^), located in the urban agglomeration of the Pearl River Delta (PRD) region, has its air quality condition different from other mega-cities in China. Due to Shenzhen’s geographical location, its air quality can reflect both the characteristics of air pollution and the transportation patterns between Hong Kong and PRD by region. Although Shenzhen’s ozone concentration is relatively lower compared with other mega-cities, ozone pollution is the primary air pollutant in Shenzhen while other mega-cities in China take PM_2.5_ as the primary air pollutant according to the environmental monitoring data [2, 19]. So, Shenzhen is selected as the typical research region in this work, for the purpose of detecting the impact of inhaled ozone on respiratory diseases. Few previous studies have been carried out in this region, and almost all of them estimate the relative risk (RR) of ozone for certain disease based on the lag effect of air pollution on respiratory diseases [19–21]. However, statistical relevance does not mean the causative influence between ozone and respiratory hospitalizations. This study is intended as a causative analysis for China to strengthen the evidence on ozone exposure and hospitalizations for respiratory diseases.

The purpose of this article is to investigate the impact of ambient ozone on respiratory diseases in a specific mega-city of China. Traditional GAM and a new convergent cross mapping method (CCM) are performed to examine the relationship and the causality influence between ozone and respiratory hospitalizations.

## Data and methods

### Data collection

**Fig. 1:**
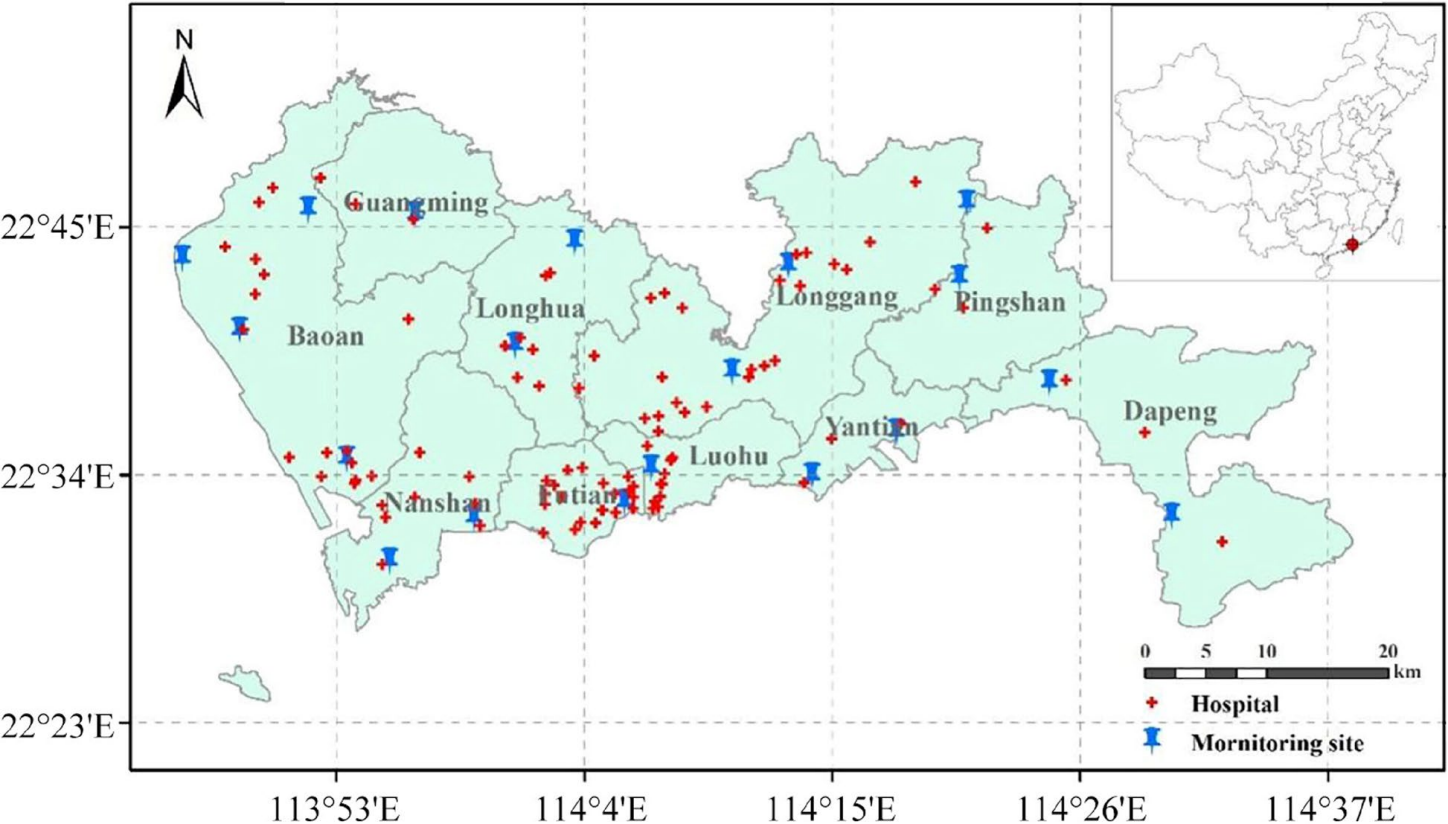
The geographical distribution of Shenzhen’s hospitals and monitoring stations

Hourly concentrations of real-time ozone (unit, µg/m^3^) from January 1st to December 31st in 2013 at 19 ambient air quality monitoring stations in Shenzhen (Fig. 1) are released by the Ministry of ecology and environment of the People’s Republic of China. Here, the daily-averaged mass concentration of ozone is calculated and days with instrument failure are excluded. Finally, efficacious (the proportion of missing data is less than 1%) monitoring samples are obtained on 349 days.

Daily emergency hospital admissions for acute respiratory diseases are collected from the Shenzhen Center for Disease Control and Prevention. Every case includes the date, hospital name, gender, age, and medical diagnosis of the disease. The geographical distribution of hospitals in Shenzhen is shown in Fig. 1. The respiratory diseases are defined according to the 10th revision of the International Classification of Diseases from J00-J99 and the following categories are considered in this study: (1) acute upper respiratory diseases (AURD, J00-J06), (2) acute lower respiratory diseases (ALRD, J20-J22). The analysis focuses on different genders and ages.

Because detailed information on the patient’s address is not available, we cannot assign a unique ozone concentration to each patient. On the assumption that people are more possible to visit a hospital nearby, we assign the monitored concentration of ozone to patients according to their proximity to the hospitals.

The annual average ozone concentration is 70 µg/m^3^, ranging from 19 to 207 µg/m^3^. Among 109,451 hospital admissions for total respiratory disease, there are 11,855 visits for AURD and 23,093 visits for ALRD. The average daily number of hospital visits for total respiratory disease is 314 and 60.1% of which are male patients (Table 1).

**Table 1:** Statistics of the main health outcomes and sub-categories by age groups and gender

		Total respiratory disease	AURD	ALRD
	**Total**	109,451	11,855	23,093
Gender	**Male**	65,821	7106	14,327
	**Female**	43,630	4749	8766
Age Group	**0–14**	74,150	10,503	19,217
	**15–64**	24,662	1196	2981
	**65-**	10,639	156	895

### Statistical analysis model for time series study

In this research, a GAM with log link and Poisson error function is applied to explore the relationship between daily ozone and respiratory disease admissions. Before the model analyses, two steps should be done: firstly, develop the best base and the main model (without or with a pollutant), and then add ozone concentration to the final model, assuming that the relationship between ozone concentration and the logarithmic respiratory hospitalizations is linear.

First, the basic model which excludes ozone is set up. The natural cubic smoothing spline function (*ns*) of calendar time is included, allowing for a non-linear relationship with respiratory hospitalization. The best degrees of freedom (*df*) for the spline calendar time are determined by the partial autocorrelation function. The best *df* for calendar time is determined through Akaike Information Criterion (AIC). In order to control seasonal and long-term trends, an *ns* (*df* = 7) is applied. The day of the week (*DOW*) is also used as a categorical variable. After the establishment of the basic model, we introduce ozone concentration and analyze its association with hospitalization. The final model can be expressed as:

$$\text{log}\left[\text{E}\left({\text{Y}}_{t}\right)\right]={\upalpha }+ {{\upbeta }}_{1}{{Ozone}}_{t-i}+ns\left(\text{T}\text{i}\text{m}\text{e},df=7\right)+{{\upbeta }}_{2}DOW$$

where $E\left({Y}_{t}\right)$ denotes the expected number of daily respiratory hospital admissions on day *t*, ${\upalpha }$ the intercept term; ${{\upbeta }}_{1}$ the regression coefficient of the log-relative rate of respiratory hospitalization related with the increase in ozone; ${Ozone}_{t-i}$ the mean ozone concentration on day *t*, *i* the day lag, and $ns\left(\text{T}\text{i}\text{m}\text{e},df=7\right)$ the smoothing function of calendar time. A detailed introduction of GAM can be found in Wood’s book [22].

The exposure-response relationship is analyzed using the smoothing function in GAM to verify the linear hypothesis of log-relative risk of respiratory hospitalizations with the concentration of ozone. The linear effects of ozone for the current day and up to two weeks prior to the outcome (lag0 to lag14) are then estimated. Effects are examined for each age group (0–14 years, 15–64 years, and >65 years) and gender to identify the most vulnerable group [23]. The statistical significance (Z-test) of gender or age differences is tested by calculating$\left({\delta }_{1}-{\delta }_{2}\right)/\sqrt{{SE}_{1}^{2}+{SE}_{2}^{2}}$, where ${\delta }_{1}$ and ${\delta }_{2}$ are coefficients for the two categories to be compared, and SE_1_ and SE_2_ stand for the respective standard errors [24].

The analyses in this study are performed using the R packages “DLM” and “MGCV”. The results are expressed as the relative risk (RR) and its 95% confidence interval (CI) in the daily hospitalizations per 10 µg/m³ increase in ozone concentration.

### CCM method

It is well-known that correlation does not equal to causality and CCM is well suited to detect mirage correlation and reveal underlying causality, as proved in complex ecosystems studies [18, 25, 26]. CCM is based on Takens’ theorem, requiring only mild assumptions. Based on it, only by using a time series with one single variable, the dynamics of high dimensional systems can be reconstructed [27]. Taking the theoretical variables of the two time series X{x_1_,x_2_,…,x_L_} and Y{y_1_,y_2_,…y_L_} (L is the length) into account, the shadow manifold Mx and My are firstly reconstructed according to the lagged-coordinate vectors X, Y, as follows,

$$x\left(t\right)=\left\langle x_t,x_{t-\tau},x_{t-2\tau},\dots,x_{t-\left(E-1\right)\tau}\right\rangle$$

$$y\left(t\right)=\left\langle y_t,y_{t-\tau},y_{t-2\tau},\dots,y_{t-\left(E-1\right)\tau}\right\rangle$$

where $t=1+\left(E-1\right)\tau$ to t = L, *E* the embedding dimension and $\tau$ a positive time lag. Then, a cross-mapped estimation of y_t_ is created, (${\widehat y}_t\left|M_x\right)$, and the nearest neighbors in ${M}_{y}$ are computed with a weighted mean, which is determined by:

$${w}_{i}=\frac{{u}_{i}}{\sum _{j=1}^{E+1}{u}_{j}}$$

where,

$${u}_{i}=\text{e}\text{x}\text{p}\left(-\frac{d[x\left(t\right),x({t}_{i}\left)\right]}{d[x\left(t\right),x({t}_{1}\left)\right]}\right)$$

The *y*_*t*_ estimation forms a locally weighted mean of the *E + 1*, as:

$${\widehat y}_t\vert M_x=\sum_{i=1}^{E+1}w_iy_{t_i}$$

The Pearson correlation coefficient between original and estimated time-series can be expressed as:

$$\rho_{Y\widehat Y}=\rho\left(y_t,{\widehat y}_t\vert M_x\right)$$

At a level of significance, a t-statistic of the correlation coefficient can be described as:

$$t= \frac{{\rho }_{Y\widehat{Y}}}{{s}_{\rho }} where\;{s}_{\rho }= \sqrt{\frac{1-{\rho }_{Y\widehat{Y}}^{2}}{N-2}}$$

where *N* denotes the length of the time series and ${\rho }_{YX}$ can be used as an indicator for the degree of influence of the *Y* dynamics on the *X* dynamics. The higher the ${\rho }_{YX}$, the stronger the causative influence is. It is crucial to optimize *E* and *τ* tuning parameters, in this research, *E* is set to 2 using the false nearest neighbor method, and *τ* equals 2, according to the average mutual information criterion.

## Results and discussions

### Temporal variation of ozone and respiratory infection

**Fig. 2:**
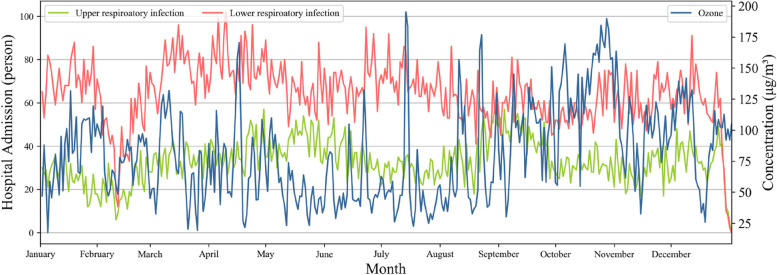
Temporal variations of daily number of patients and ozone concentration in Shenzhen in 2013

The temporal pattern of daily patients and ozone concentration in Shenzhen in 2013 is shown in Fig. 2. The maximum and minimum values of ozone appeared in October and July with mean values of 143.85 ± 27.75 µg m^-3^ and 53.02 ± 38.19 µg m^-3^, respectively. The concentration of ozone in May (54.41 ± 21.27 µg m^-3^) was very close to the value in July. The daily count of respiratory hospitalizations ranged from 0 to 177, and its inter-annual variation indicated that the lowest hospital admission folks during 2013 was in February for both of upper and low respiratory infections. The high prevalence rate of upper respiratory infection was mainly in May and September, relating to the warm season from the statistical distribution. The temporal trend of lower respiratory infection, except the valley value in February, is much steady with litter fluctuation, and relatively high hospital admission is primarily around March and April. The monthly average number of respiratory patients was 2973 (range from 1969 to 3525), and 61.43% (range from 59.65 to 62.82%) were male patients. Children (age from 0 to 14) were the principal part, accounting for 85.17% (80.45–88.76%) of total patients. The correlation analyses indicate that hospital admissions almost have no correlation to the ozone with a Pearson correlation coefficient ranging from − 0.06 to 0.1 (for different age and gender) and from − 0.13 to 0.05 (for different age and gender) for upper and lower respiratory infections, respectively.

### Association between ozone and respiratory hospitalization

**Fig. 3:**
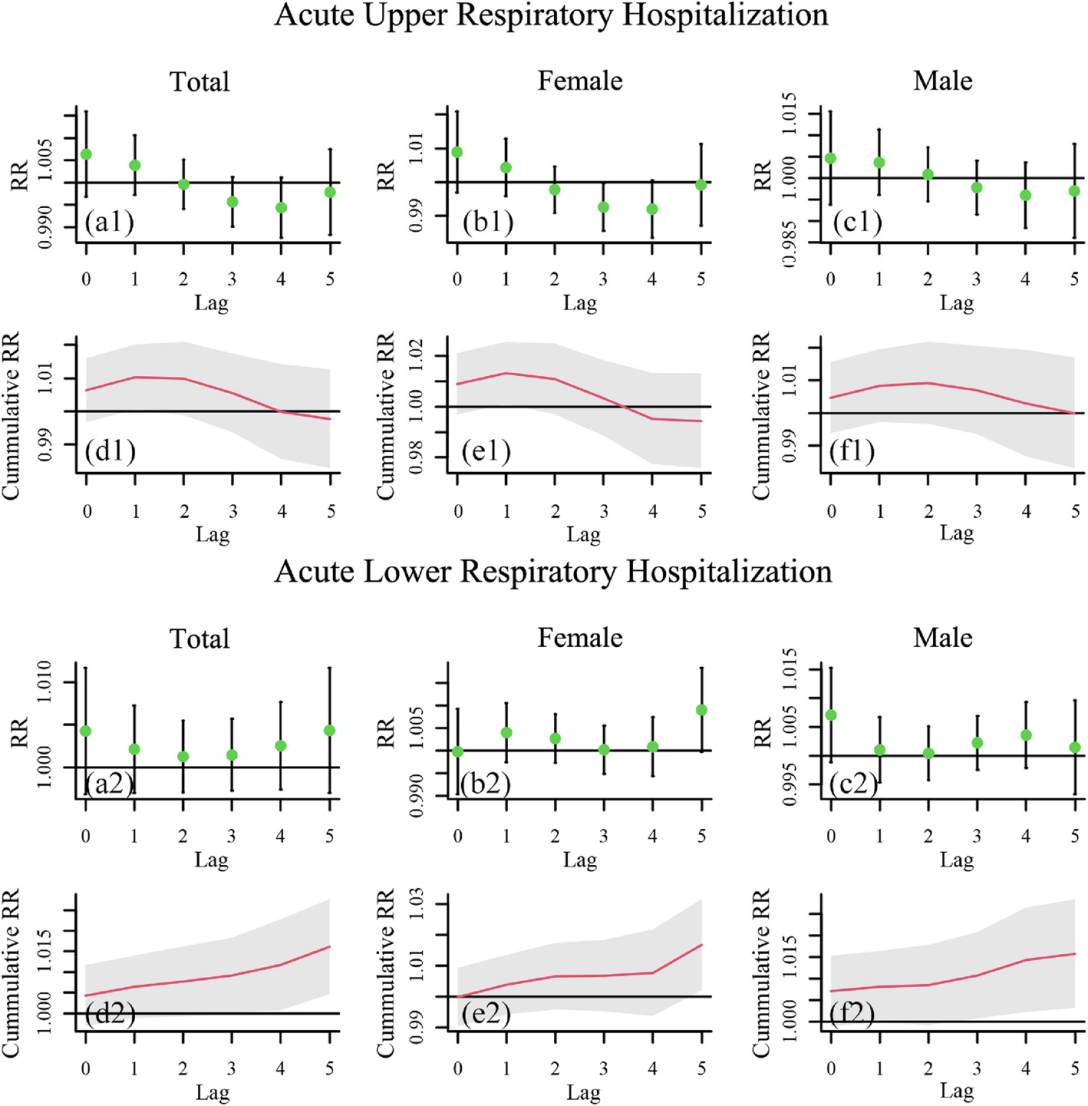
Lag-response patterns of RR of single-day and cumulative lag effect on different gender groups with a lag of 5 days (a1 − f1) for acute upper respiratory hospitalization, (a2 − f2) for acute lower respiratory hospitalization

**Fig. 4:**
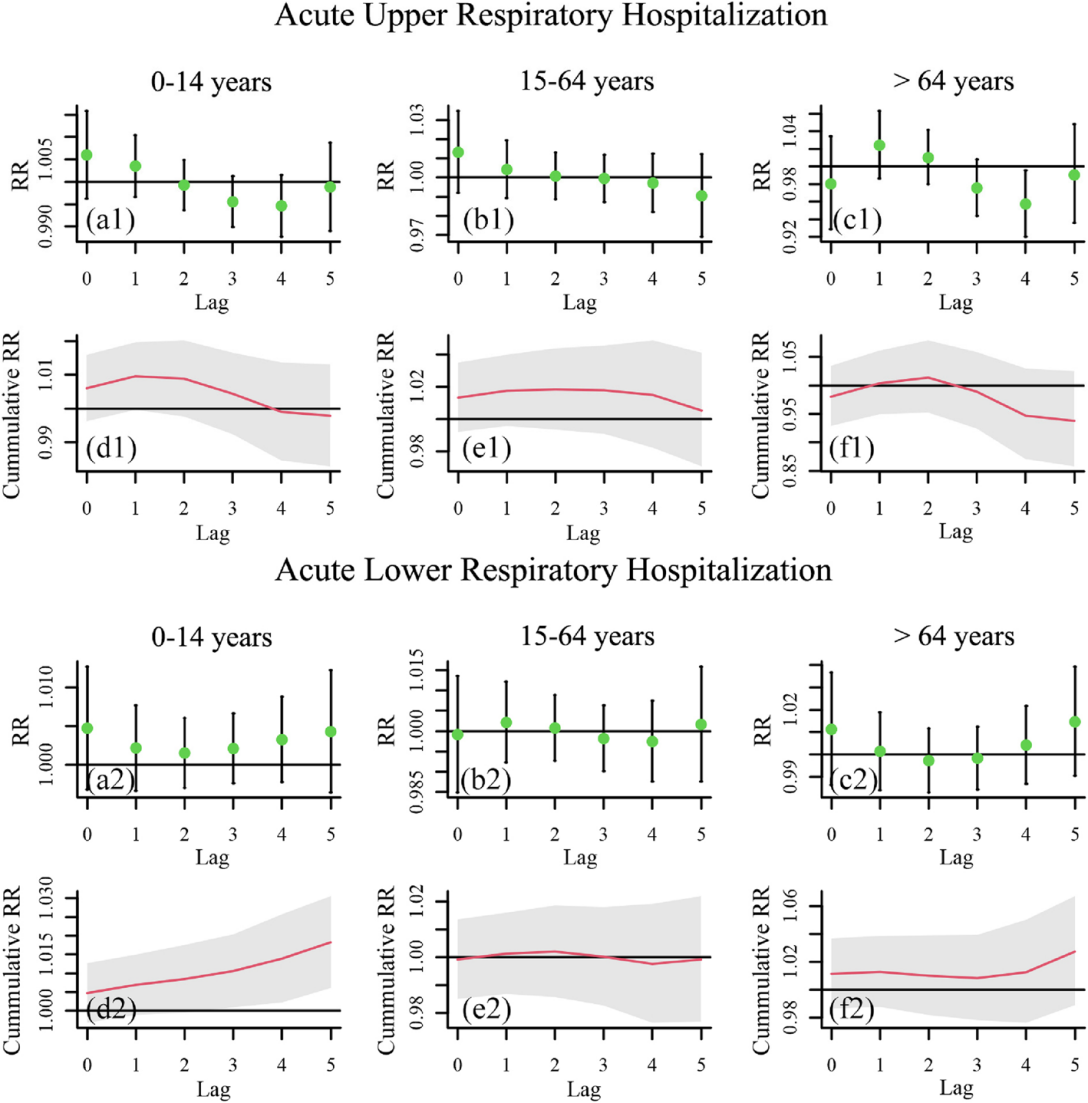
Lag-response patterns of RR of single-day and cumulative lag effect on different age groups with a lag of 5 days (a1 − f1) for acute upper respiratory hospitalization, (a2 − f2) for acute lower respiratory hospitalization

To explore the lagged health impact of O_3_ on different population groups, including male group, female group, and different age groups, a time series analysis using GAM was conducted in terms of acute respiratory infections. The resulting lag-response patterns of RR of single-day and cumulative lag effect with a lag of 5 days for different population groups are shown in Figs. 3 and 4 (95% CI: black bars and grey areas). The result is considered to be significant if the confidence interval for RR/Cumulative RR and the horizontal line RR/Cumulative RR = 1 do not intersect. Generally, O_3_ exhibits a significant lag effect on acute lower respiratory hospitalizations (ALRHs) of the total population from a lag of 4 days in cumulative mode, and for each 10 µg/m³ increase in O_3_, the number of hospitalizations of total population due to ALRHs would generally increase 1.6% (95%CI: 0.5%, 2.8%) within a lag of 5 days. The numerical single-day (lag0 to lag5) and accumulative (Lag05) RR values for hospitalizations of different population groups corresponding to a 10-unit increase in O_3_ within 5 days, together with its 95% CI, are shown in Tables 2 and 3.

**Table 2:** Numerical single-day and cumulative RR for hospitalizations of different gender groups corresponding to a 10-unit increase in O_3_ within 5 days, together with its 95% CI (*P < 0.05)

LAG	Acute Upper Respiratory Hospitalization			Acute Lower Respiratory Hospitalization		
	Total	Female	Male	Total	Female	Male
Lag0	1.006 (0.997,1.016)	1.004 (0.997,1.011)	1.000 (0.994,1.005)	1.004 (0.997,1.012)	1.002 (0.997,1.007)	1.001 (0.997,1.006)
Lag1	1.009 (0.997,1.021)	1.004 (0.996,1.013)	0.998 (0.991,1.005)	1.000 (0.99,1.009)	1.004 (0.997,1.011)	1.003 (0.997,1.008)
Lag2	1.005 (0.994,1.016)	1.004 (0.996,1.011)	1.001 (0.995,1.007)	1.007 (0.999,1.015)	1.001 (0.995,1.007)	1.000 (0.996,1.005)
Lag3	1.006 (0.996,1.016)	1.004 (0.997,1.010)	0.999 (0.994,1.005)	1.005 (0.997,1.013)	1.002 (0.997,1.008)	1.002 (0.997,1.006)
Lag4	1.013 (0.992,1.035)	1.004 (0.989,1.019)	1.001 (0.989,1.013)	0.999 (0.985,1.014)	1.002 (0.992,1.012)	1.001 (0.993,1.009)
Lag5	0.980 (0.929,1.034)	1.024 (0.986,1.063)	1.010 (0.98,1.041)	1.011 (0.986,1.037)	1.001 (0.984,1.019)	0.997 (0.983,1.012)
Lag05	0.998 (0.981,1.013)	0.994 (0.976,1.013)	0.999 (0.983,1.017)	1.016^*^ **(1.005,1.028)**	1.017^*^ **(1.002,1.032)**	1.016^*^ **(1.003,1.029)**

In terms of different gender groups (Fig. 3), significant lag effect is detected for both male group and female group in cumulative mode with different lag days, i.e., a lag of 5 days for female group and a lag of 3 to 5 days for male group. For each 10 µg/m³ increase in O_3_, the number of hospitalizations of male group and female group due to ALRHs would generally increase 1.6% (95%CI: 0.3%, 2.9%) and 1.7% (95%CI: 0.2%, 3.2%) respectively, within a lag of 5 days. In terms of different age groups (Fig. 4), it is observed that O_3_ only has a significant lag effect on ALRHs of children from 0 to 14 years in a lag of 3 to 5 days in cumulative mode. As for acute upper respiratory hospitalizations (AURHs) of all age groups, and ALRHs of people aged above 14, no significant lag effects are detected. For each 10 µg/m³ increase in O_3_, the number of hospitalizations of children (0 − 14 years) due to ALRHs would generally increase 1.8% (95%CI: 0.6%, 3.1%) within a lag of 5 days.

**Table 3:** Numerical single-day and cumulative RR for hospitalizations of different age groups corresponding to a 10-unit increase in O_3_ within 5 days, together with its 95% CI (*P < 0.05)

LAG	Acute Upper Respiratory Hospitalization			Acute Lower Respiratory Hospitalization		
	0–14	15–64	> 64	0–14	15–64	> 64
Lag0	0.996 (0.99,1.001)	0.994 (0.988,1.001)	0.998 (0.988,1.008)	1.001 (0.997,1.006)	1.003 (0.997,1.008)	1.004 (0.997,1.012)
Lag1	0.993 (0.986,1.000)	0.992 (0.983,1.000)	0.999 (0.987,1.011)	1.000 (0.995,1.006)	1.001 (0.994,1.007)	1.009 (1.000,1.018)
Lag2	0.998 (0.992,1.004)	0.996 (0.988,1.004)	0.997 (0.986,1.008)	1.002 (0.998,1.007)	1.004 (0.998,1.009)	1.001 (0.993,1.010)
Lag3	0.996 (0.990,1.001)	0.995 (0.988,1.002)	0.999 (0.989,1.009)	1.002 (0.998,1.007)	1.003 (0.998,1.009)	1.004 (0.996,1.012)
Lag4	1.000 (0.987,1.012)	0.997 (0.982,1.012)	0.990 (0.969,1.012)	0.998 (0.990,1.006)	0.997 (0.988,1.007)	1.002 (0.988,1.016)
Lag5	0.976 (0.944,1.008)	0.957 (0.92,0.996)	0.990 (0.936,1.048)	0.998 (0.984,1.012)	1.004 (0.987,1.022)	1.015 (0.990,1.039)
Lag05	0.998 (0.983,1.013)	1.005 (0.971,1.041)	0.937 (0.858,1.025)	1.018^*^ **(1.006,1.031)**	1.000 (0.977,1.022)	1.027 (0.989,1.067)

### Causality influence of ozone on respiratory hospitalization

The causative impact of ozone on regional hospitalization for respiratory diseases is quantified using CCM. Because CCM involves convergence, with the time-series length *L* increases, the estimation skill of cross-mapping estimates improves. This is an important distinguishing property between causation and simple correlation. Causality is only found when this pattern is present, otherwise, there is no causal relationship between the two variables. The strength of the effect is determined by the ρ value of predictive skill.

**Fig. 5:**
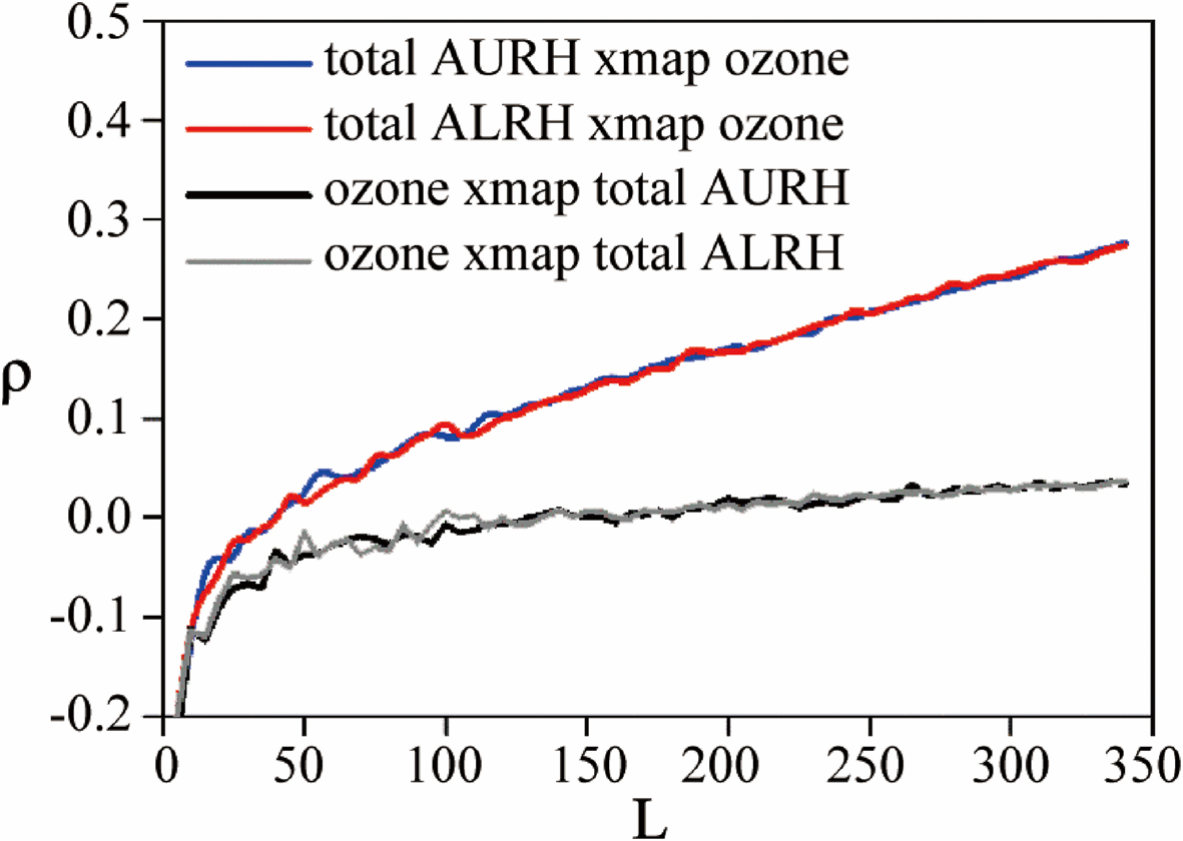
Validation of the CCM to the causative analyses of the ozone influence to the respiratory patients

Figure 5 presents the convergent cross map of one kind of classification scenario. The blue solid line and red line both show the pattern mentioned above, indicating that ozone concentration has a significant effect to the upper and lower respiratory. In contrast, the gray solid line and black solid line are not, which means the number of cases of the mentioned respiratory diseases has no causal effect on ozone concentration. This result validates the feasibility and superiority of the CCM method. Figure 5 demonstrates that inhaled ozone has a significant causative impact on hospitalization rates of both the upper and the lower respiratory diseases. Whether this phenomenon is related to the sedimentation velocity of ozone in the respiratory system and the effect of a stimulus on the local mucosa still needs to be further discussed with more data and case-control studies.

**Fig. 6:**
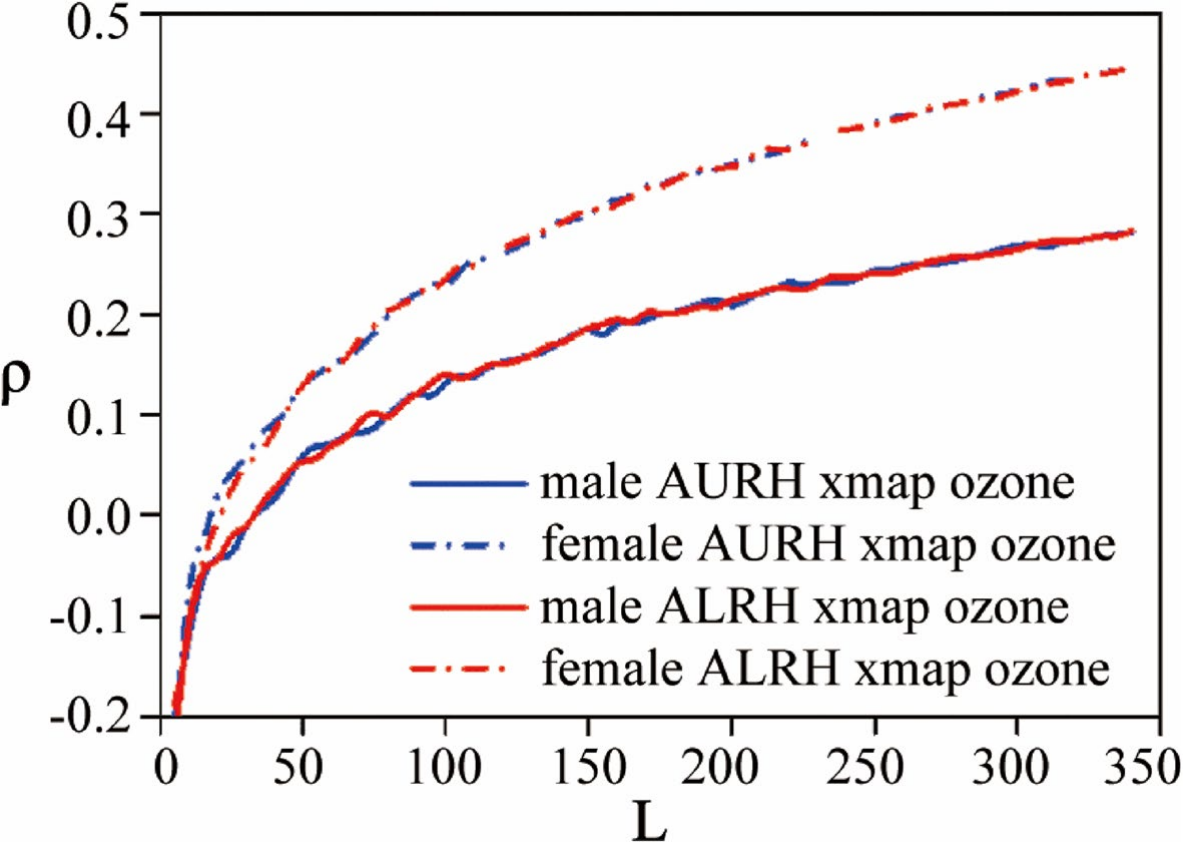
Convergent cross maps illustrating the causative impact of inhaled ozone concentration on AURH and ALRH for different genders

Furthermore, the harmful effects of ozone to human health vary with gender and age (Fig. 6). It can be seen that females are more susceptible to inhaled ozone than males. Previous study also found that females are more affected by short-term ozone exposure [28], and this finding is consistent with the animal experiments, in which female rats are more prone to ozone effects than male rats [19]. The sex-based difference may be relative to the estrogen level and the differential regulations of lung immune response [13, 20]. Estrogen may be important in stimulating inflammation, and lactation is related to increased secretion of proinflammatory hormones. Gunnison et al. [21] also find the increasing inflammation and injury in the lungs of pregnant or lactating rats exposed to ozone.

**Fig. 7:**
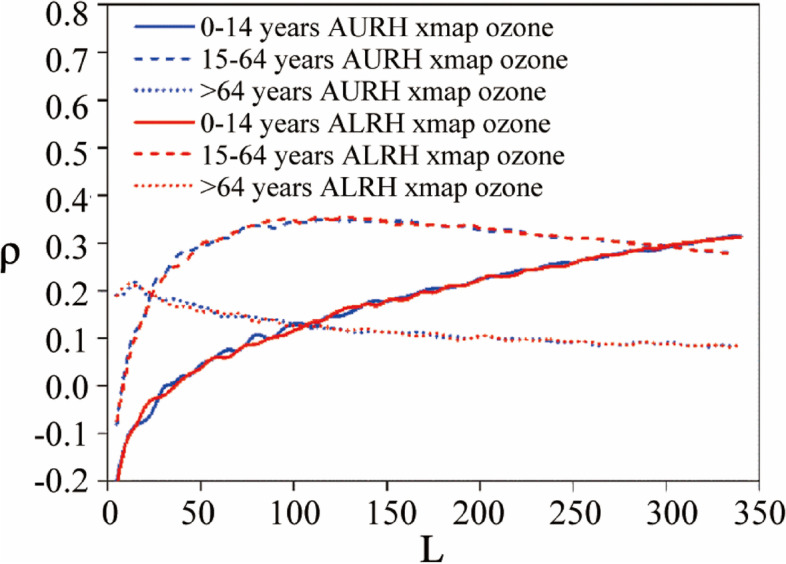
Convergent cross maps illustrating the causative impact of inhaled ozone concentration on AURH and ALRH for different ages

Besides, adults are more sensitive to ozone exposure than children (Fig. 7). It could be caused by that the tolerance to ozone poisoning is transient, and that the stress reactions to ozone of children need longer time than adults because their respiratory systems are not fully developed. This phenomenon is also proved by measuring changes in total protein multiplicity in bronchoalveolar lavage fluid, where ozone-induced lung injury in neonatal mice is less sensitive than in adult mice [15, 29, 30]. Moreover, the relative long-term exposure of adults also could be also attributed to much more infectibility of adults.

Figure 7 also demonstrates that the elderly are more tolerant to ozone exposure than adults and children, although respiratory infection is a common cause of acute hospitalization for the elderly. In fact, the dynamic lung capacity of the elderly, such as forced vital capacity and forced expiratory volume in 1 s, commonly decreases, and the decline of pulmonary ventilation function for the elderly shows a significant increase with the increase of age. So, the hospitalization rate of the acute respiratory diseases of the elderly is probably related to pulmonary hypofunction and has little correlation with ozone inhalation.

Compared with other mega cities in China, Shenzhen has a relatively lower level of ozone pollution. For example, Shenzhen’s annual average concentration of O_3_ was 70 µg/m^3^ in 2013, while the annual average O_3_ concentration in Beijing was 84.75 µg/m^3^ in the same year. Our results also find that even relatively low levels of ozone exposure are significantly associated with hospitalization for acute respiratory diseases, which is the same as the findings in previous study [31].

## Conclusions

The core objective of this study is to detect the causative impact of ozone on the human respiratory system. Shenzhen, a typical city, is selected as the study area because ozone is the primary air pollutant here. Daily concentration of ozone and hospital admissions of respiratory infections in 2013 are used in this study. The lag-time correlation analysis (GAM) and the causal detection method (CCM) are utilized to quantify the lag effect and the causative influence of ozone on respiratory hospitalizations.

The relationship between ozone and respiratory sickness is a complicated dose response relation. Our analysis shows that traditional lag-time correlation analysis is hard to detect the correlation of ozone with respiratory hospitalization, and fails to reveal the influence of ozone on respiratory hospitalization. In contrast, CCM suggests that ozone has a significant causative impact on both AURHs and ALRHs. Meanwhile, the causative impacts vary with gender and age. The quantitative analysis results (ρ) of CCM manifest that ozone has a more significantly causal influence on female acute respiratory than male. Adults are more sensitive to ozone exposure than children, and the elderly are more tolerant than adults and children. The significant causative influence of ozone on respiratory hospitalization should be paid more attention. Still, there are some limitations in this study. The ozone concentration cannot be measured for each patient, due to the lack of information about the patient’s address. In this study, we analyze the relationship between ozone concentration and hospitalization for acute respiratory diseases in Shenzhen in 2013, and this relatively short time series may bring certainties to our analysis results. Longer time series studies and more studies in other areas are expected in the future.

## Data Availability

The hourly concentration data of real-time ozone used and analyzed during the current study is available in the Ministry of ecology and environmental of the people’s republic of China repository (https://www.mee.gov.cn/). The daily hospital admission anonymized data for respiratory diseases is available from https://github.com/Lstarry/EResDATA.git.
